# Research and Remedies

**Published:** 1912-10-12

**Authors:** 


### RESEARCH AND REMEDIES.

### Subcutaneous Cricotomy.

An interesting and admirably brief report of a case of laryngeal stenosis following intubation in diphtheria is given in the *Australasian Medical Gazette* by Drs. Croll and Turner. The patient was a girl, six years of age, and was seen on the fourth day of a laryngeal diphtheria, deeply cyanosed and almost *in extremis*. Intubation gave temporary relief; but the smaller sizes of tube were repeatedly coughed out, and the big one used instead set up laryngeal ulceration and stenosis. The parents were much distressed at the idea of a tracheotomy scar; so Dr. Turner divided the cricoid cartilage subcutaneously with a tenotomy knife. After this an intubation tube could be worn fairly comfortably, but it was two months before it could be dispensed with entirely. During this period constant care and attention were required to maintain dilatation of the stricture. The successful result was, therefore, well deserved; and the novel operation should certainly be made a note of by fever hospital medical officers.

### Renal Infantilism.

A good deal of attention has been paid lately by pædiatrists to infantilism and arrested development generally. In the *British Journal of Children's Diseases* Drs. Miller and Parsons describe certain types of this condition, which are associated either with actual chronic kidney disease or with diabetes insipidus; they adopt the term "renal infantilism" to cover the whole group. They hold also that the infantilism is secondary to the perversion of the renal functions. When organic kidney disease exists, it is usually non-syphilitic chronic interstitial nephritis; the degree of infantilism is severe and death is common in childhood from uræmia or pneumonia. The other type, associated with diabetes insipidus, is often due to inherited syphilis, organic nervous lesions, or other more indefinite causes. The infantilism is less severe in grade, and life may be prolonged. In both classes the symptoms may be present from birth, or may develop later on in early childhood. No treatment hitherto tested has had any effect upon any case of either type.

### Two Elegant Prescriptions.

Dr. B. Bramwell has two formulæ for rendering less nauseous the combination of potassium iodide and perchloride of mercury so often prescribed in tertiary syphilis. The first is for those who prefer sweet flavourings, the second for those to whom bitters appeal more strongly.

| | | |
|---|---|---|
| (*a*) | Liq. hydrarg. perchlor. ... ... ... | ℥j. |
| | Potass. iodidi ... ... ... ... ... | ʒj. |
| | Syrupi ... ... ... ... ... ... | ʒj. |
| | Mucil. tragac. ... ... ... ... | ℥ij. |
| | Ol. amyg. essen. (sine HCN) ... ... | ♏ij. |
| | Ol. cinnamomi ... ... ... ... | ♏ij. |
| | Aq. chlorof. ad ... ... ... ... | ℥viij. |
| | An eighth part for a dose. | |
| (*b*) | Liq. hydrarg. perchlor. ... ... ... | ℥j. |
| | Potass. iodidi ... ... ... ... ... | ʒj. |
| | Tr. chirettæ ... ... ... ... ... | ʒiss. |
| | Elixiris glucidi ... ... ... ... | ♏xl. |
| | Inf. gent. co. ad ... ... ... ... | ℥viij. |
| | An eighth part for a dose. | |

